# Changes in leisure time physical activity and subsequent disability retirement: A register-linked cohort study

**DOI:** 10.1186/s12966-016-0426-2

**Published:** 2016-09-06

**Authors:** Jouni Lahti, Ansku Holstila, Minna Mänty, Eero Lahelma, Ossi Rahkonen

**Affiliations:** Department of Public Health, University of Helsinki, P.O. Box 20, Helsinki, FIN-00014 Finland

**Keywords:** Exercise, Physical inactivity, Work disability, Working conditions

## Abstract

**Background:**

Disability retirement is an economic, public health and work life issue causing costs for employees, workplaces and society. Adopting physical activity at middle-age has been associated with reduced risk of sickness absence and mortality. The aim of this study was to examine how changes over time in leisure time physical activity are associated with subsequent disability retirement among midlife employees.

**Methods:**

The Helsinki Health Study cohort baseline (phase 1) mail questionnaire survey data were collected in 2000, 2001 and 2002 among 40–60-year-old employees of the City of Helsinki, Finland. A phase 2 survey was conducted in 2007 (*N* = 3943). Respondents were classified into three groups: 1. low-active (<14 MET-hours/week), 2. moderately active (> = 14 MET-hours/week in moderate-intensity physical activity) and 3. vigorously active (> = 14 MET-hours/week including vigorous physical activity) at both phases. This yielded nine groups for describing stability and change of leisure time physical activity. Disability retirement data were derived from the registry of the Finnish Centre for Pensions until the end of 2013. A Cox regression analysis was used to calculate hazard ratios (HR) and their 95 % confidence intervals (CI) adjusting for covariates.

**Results:**

During the follow-up, 264 (6.7 %) participants retired due to disability. Compared with those who were persistently low-active, those who increased their physical activity from low to vigorous had a lower risk of subsequent disability retirement (HR = 0.38, 95 % CI = 0.15–0.97) when adjusting for age, gender, occupational social class, strenuousness of work, smoking and binge drinking. Similarly, compared with those who were persistently moderately active, those increasing from moderate to vigorous (HR = 0.50, 95 % CI = 0.28–0.86) had a reduced risk. In contrast, those decreasing their physical activity from vigorous to low (HR = 2.42, 95 % CI = 1.32–4.41) or moderate (HR = 1.70, 95 % CI = 1.03–2.82) had an increased risk, compared with those who were persistently vigorously active. Adjusting for BMI, limiting longstanding illness and prior sickness absence somewhat attenuated the associations.

**Conclusions:**

Adopting vigorous physical activity was associated with a reduced risk of disability retirement. Promoting vigorous physical activity among midlife employees may help prevent disability retirement.

## Background

Early retirement due to disability is a major economic, public health and work life problem in many Western countries, causing costs for employees, workplaces and society [1]. Previous studies have shown that healthy behaviours including a sufficient amount of leisure time physical activity are associated with a lower risk of disability retirement [2, 3]. Our earlier study suggested that especially vigorous physical activity is associated with a reduced risk of disability retirement [4]. Previous studies on various health outcomes such as metabolic syndrome [5], sickness absence [6] and mortality [7–9], also suggest that vigorous activity may provide additional health benefits over those provided by physical activity of moderate intensity.

Adopting physical activity during midlife contributes to a reduced risk of major chronic diseases [10] and mortality [11], as well as to a better health-related quality of life later on [12]. Furthermore, our previous studies have shown a reduced risk of sickness absence among those adopting vigorous physical activity during midlife [13]. The reduced risk concerns especially sickness absence due to musculoskeletal causes [14]. A prior study examined changes in health behaviours, including physical activity, between two surveys 25 years apart among Swedish twins, but the associations with disability retirement were only weak [15]. Permanent work disability usually takes several years to develop and those with health problems may also face limitations with engaging in physical activity, especially vigorous activity. In order to establish causal associations between physical activity and disability retirement, the previous health status needs to be considered. In addition to health measures, we also included key covariates in the analyses that are known risk factors for disability retirement such as smoking and alcohol use [15], strenuousness of work and socioeconomic position [16] as well as body mass index [15], which may also be unequally distributed between physical activity groups [17, 18].

We aimed to examine how changes in leisure time physical activity are associated with the risk of subsequent disability retirement. Based on previous studies, we expected that adopting or increasing physical activity is associated with a reduced risk and decreasing physical activity with an increased risk of disability retirement. In addition, we expected that the associations are stronger regarding changes in vigorous activity.

## Methods

### Study population

The Helsinki Health Study cohort phase 1 surveys were collected in 2000, 2001 and 2002. The mail questionnaires were sent to employees of the City of Helsinki, Finland, who reached the ages of 40, 45, 50, 55 and 60 years during each survey year [19]. The sample consisted of 13,346 persons. The response rate at baseline was 67 % (*n* = 8960). The phase 2 survey data were collected in 2007 from the respondents who participated in the baseline survey, with a response rate of 83 % (*n* = 7332). Of the respondents, 80 % were women, corresponding to the target population and the Finnish municipal sector in general. Identical questions on leisure time physical activity and covariates were applied in both surveys. The questionnaire survey data were individually linked to the registry of the Finnish Centre for Pensions until the end of 2013 using unique personal identification numbers. Of the phase 2 respondents, 76 % (*N* = 5575) gave written consent for the linkage. We excluded those who retired (*n* = 1347) or were otherwise unemployed (*n* = 155) before the phase 2 survey. In addition, we excluded respondents who reached the age of 63 by the phase 2 survey (*n* = 27) because at that age, disability retirement is no longer granted in Finland. There was missing information on some study variables (*n* = 103) and 3943 respondents were available for the present analyses. According to non-response analyses, the data satisfactorily represent the target population and attrition, as well as declining the register linkage, are unlikely to cause major bias to the analyses [19, 20].

### Leisure time physical activity

Weekly hours of leisure time physical activity (commuting included) within the previous 12 months were assessed. Four intensity grades were given and each intensity grade was exemplified with typical activities that people usually engage in: walking, brisk walking, jogging, and running, or their equivalent activities. First, the participants were instructed to estimate the intensity of their physical activities. Then, they were instructed to estimate their average weekly hours of physical activity in each intensity grade ranging from zero to more than 4 h per week. Based on their responses, leisure time physical activity was converted to an approximate metabolic equivalent (MET) index. MET-hours per week for leisure time physical activity were calculated by multiplying the time spent by the estimated MET value of each intensity grade [21] and summing up the four values [22]. Based on a classification used in our previous study [13], the respondents were classified into three groups in both surveys: 1. low-active (under 14 MET-hours/week), 2. moderately active (at least 14 MET-hours/week in moderate-intensity physical activity only such as walking/brisk walking) and 3. vigorously active (at least 14 MET-hours/week, including vigorous physical activity such as jogging/running). This yielded nine groups, describing transitions from one leisure time physical activity group to another. For example, 15 MET-hours corresponds to 2,5 h of brisk walking, which is recommended for health benefits [23].

### Disability retirement

The Finnish disability retirement scheme includes all granted (permanent, temporary and partial) disability pensions that require a medically confirmed illness or disease that essentially reduces working capacity or prevents working in the person’s occupation [24]. For descriptive purposes, we distinguish the two main medical causes for disability retirement according to the ICD-10 classification: musculoskeletal (ICD codes M00-M99) and mental (F00-F99) causes. The follow-up started on the day the phase 2 questionnaire was returned and continued until the end of the year 2013 or on the date of other form of retirement, death or the age of 63 years (after which, disability pensions are no longer applicable in Finland). The mean follow-up time was 6.0 years.

### Covariates

Covariates included age, gender, and occupational social class, physical and mental strenuousness of work, smoking, binge drinking, body mass index (BMI), limiting longstanding illness (LLI) and prior sickness absence. Age was categorised into five groups by phase 1 age 40, 45, 50, 55 and 60. Occupational social class in phase 2 was classified into managers and professionals (e.g. administrators, teachers and doctors), semi-professionals (e.g. nurses and foremen), routine non-manual employees (e.g. clericals and other non-professionals), and manual workers (e.g. bus drivers and cleaners) [19]. The respondents were asked how strenuous their work is physically and mentally, with four response alternatives: ‘very light’, ‘fairly light’, ‘fairly heavy’, and ‘very heavy’. Based on these responses physical and mental strenuousness of work were dichotomised as strenuous and non-strenuous in phase 2. Smoking was categorised based on daily smoking in phase 1 and phase 2, which yielded four groups describing changes and persistency in smoking. A similar procedure was conducted for binge drinking (≥6 doses at least once a week), being over-weight (BMI ≥ 25 kg/m^2^) and LLI. The participants were also asked whether they have a longstanding illness, and if so, whether the illness restricts working or other daily tasks. The participants who reported that they have a longstanding illness that restricts their daily tasks were categorised as those with LLI, and the others were considered as being without LLI. Prior sickness absence was derived from the registries of the Social Insurance Institution of Finland. Sickness absence over 10 working days during the 12 months prior to the phase 2 survey was examined [14].

### Statistical methods

A Cox regression analysis was used to calculate hazard ratios (HR) and their 95 % confidence intervals (95 % CI) for subsequent disability retirement. Persistently low, persistently moderate and persistently vigorous physical activity were used as reference groups to which we compared the changes across these groups. Men and women were pooled for the analyses. In model 1, the HR was adjusted for age and gender. In model 2, additional adjustments were made for occupational social class, strenuousness of work and changes in smoking and binge drinking. Model 2 was further adjusted for BMI (model 3), limiting longstanding illness (model 4), and prior sickness absence (model 5). Model 2 is considered the main model, adjusted for purely confounding factors, and additional adjustments are made in separate models for covariates that may also mediate the examined associations. The proportional hazards assumption was confirmed using Schoenfeld residuals [25]. The IBM SPSS 23.0 statistical package was used for the analyses.

## Results

Those who were less physically active tended to be older and from lower SEP groups compared to the vigorously active (Table 1). Physically strenuous work was less common among the persistently vigorously active, whereas, mentally strenuous work was more common among the persistently low-active. Smoking, binge drinking and overweight as well as LLI and prior sickness absence tended to be more common among the less active.

**Table 1:** Description of study variables by physical activity change groups

		Leisure time physical activity								
		Low at 2000-02			Moderate at 2000-02			Vigorous at 2000-02		
	All	Low at 2007	Moderate at 2007	Vigorous at 2007	Low at 2007	Moderate at 2007	Vigorous at 2007	Low at 2007	Moderate at 2007	Vigorous at 2007
N	3947	370	336	158	294	915	371	164	400	935
Women (%)	81.0	78.4	82.1	75.3	83.3	89.5	87.3	73.2	84.3	70.8
Age (mean)	47.3	48.4	47.9	46.2	47.4	48.3	46.5	47.0	47.8	46.2
Occupational class (%)										
Managers/professionals	32.9	30.8	28.9	40.5	26.5	26.8	32.3	34.8	34.0	41.3
Semi-professionals	20.8	18.1	22.0	22.2	21.8	17.7	24.5	17.1	19.3	23.9
Routine non-manuals	34.4	34.9	36.9	29.7	33.0	43.6	33.7	37.8	36.3	24.6
Manual workers	11.8	16.2	12.2	7.6	18.7	11.9	9.4	10.4	10.5	10.3
Physically strenuous work (%)	29.7	29.7	30.7	26.6	32.7	35.8	31.5	26.8	31.8	21.8
Mentally strenuous work (%)	12.2	18.1	13.1	12.7	12.6	10.2	10.8	14.0	12.8	11.6
Changes in smoking (%)										
Non-smokers	75.2	65.9	68.5	79.7	63.3	74.6	80.3	72.6	74.0	83.6
Quit smoking	6.3	6.2	10.4	8.2	8.2	5.6	7.3	7.3	6.8	3.7
Became smokers	1.8	2.7	1.5	1.3	2.0	1.2	2.7	2.4	1.5	1.8
Smokers	16.8	25.1	19.6	10.8	26.5	18.6	9.7	17.7	17.8	10.8
Binge drinking (%)										
Rarely or no binge drinking^a^	69.4	65.4	67.0	70.3	63.3	72.1	75.7	61.6	68.0	70.4
Decreased binge drinking	6.3	6.5	8.3	3.8	5.4	5.7	5.9	9.8	7.8	5.7
Increased binge drinking	7.5	6.5	8.3	5.7	9.5	7.0	6.2	10.4	6.3	8.1
Binge drinking	16.9	21.6	16.4	20.3	21.8	15.2	12.1	18.3	18.0	15.8
BMI (%)										
Normal weight^b^	44.7	27.0	33.6	44.9	32.3	37.2	57.4	36.6	49.5	61.1
Became normal weight	3.7	4.6	4.5	6.3	2.4	3.8	4.9	3.7	1.3	3.4
Became overweight	11.2	10.0	11.9	8.9	13.9	11.8	7.8	17.1	13.8	9.5
Overweight	40.5	58.4	50.0	39.9	51.4	47.2	29.9	42.7	35.5	26.0
LLI (%)^c^										
No LLI	70.9	58.4	72.6	72.2	66.3	64.8	74.1	73.8	72.0	80.2
Recovered	5.4	3.9	3.2	3.1	4.8	5.4	1.2	3.0	2.9	3.9
Developed LLI	16.1	20.5	13.1	14.6	20.4	18.9	13.2	17.1	19.8	11.1
Persistent LLI	9.1	15.7	10.4	10.1	10.2	11.5	7.3	7.9	5.3	5.8
Prior sickness absence (mean)^d^	0.21	0.25	0.18	0.16	0.32	0.26	0.14	0.22	0.20	0.16

^a^≥ 6 portions of alcohol more than once a month

^b^< 25 kg/m^2^

^c^Limiting longstanding illness

^d^Any sickness absences (> 10 working days)

There were 264 disability retirement events during the follow-up (Table 2). The proportion of disability retirements during the follow-up varied between the physical activity change groups. Those that were persistently low-active had the highest proportion (10.0 %) of disability retirement events whereas those who increased from low-active to vigorously active (3.2 %) clearly had a lower proportion of events. In addition, those who increased from the moderately to vigorously active group (4.0 %) and those who were persistently vigorously active (3.5 %) had a similar lower proportion of events. Those increasing from low to moderate (6.8 %), persistently moderately active (8.6 %) or decreasing vigorous to moderate (7.2 %) had only a somewhat lower proportion of disability retirement events during the follow-up. In contrast, those decreasing from vigorous to low (9.8 %) or from moderate to low (9.2 %) were similar to those in the persistently low-active group. Disability retirement events due to main causes, i.e. musculoskeletal and mental causes by changes in physical activity are presented for descriptive purposes (Table 2); due to a low number of events, we concentrated on disability retirement due to any cause in the further analyses.

**Table 2:** Disability retirement events (%) by physical activity change groups

		Leisure time physical activity								
		Low at 2000-02			Moderate at 2000-02			Vigorous at 2000-02		
	All	Low at 2007	Moderate at 2007	Vigorous at 2007	Low at 2007	Moderate at 2007	Vigorous at 2007	Low at 2007	Moderate at 2007	Vigorous at 2007
Disability retirement events (%)	264 (6.7)	37 (10.0)	23 (6.8)	5 (3.2)	27 (9.2)	79 (8.6)	15 (4.0)	16 (9.8)	29 (7.2)	33 (3.5)
Musculoskeletal causes events (%)	111 (2.8)	16 (4.3)	9 (2.7)	1 (0.6)	12 (4.1)	38 (4.2)	5 (1.3)	6 (3.7)	11 (2.8)	13 (1.4)
Mental causes events (%)	71 (1.8)	10 (2.7)	7 (2.1)	1 (0.6)	7 (2.4)	16 (1.7)	7 (1.9)	6 (3.7)	6 (1.5)	11 (1.2)

We calculated hazard ratios for disability retirement due to any cause adjusting for covariates (Table 3). No statistically significant gender interaction was found (*p* = 0.8). In model 2 adjusted for confounders, those increasing from low-active to vigorously active (HR = 0.38, 95 % CI = 0.15–0.97) had a reduced risk of disability retirement compared with those that were persistently low-active. Similarly, those increasing from moderately active to vigorously active (HR = 0.50, 95 % CI = 0.28–0.86) had a reduced risk compared with those that were persistently moderately active, whereas, those decreasing to low-active activity showed no association. In contrast, compared with the persistently vigorously active, those decreasing to low-active (HR = 2.42, 95 % CI = 1.32–4.41) or moderately active (HR = 1.70, 95 % CI = 1.03–2.82) had an increased risk of disability retirement. Adjusting for BMI, prior sickness absence and LLI attenuated the associations further and in part, the found associations lost statistical significance.

**Table 3:** Risk of subsequent disability retirement (2007–2013) by changes in physical activity. Hazard ratios and their 95 % confidence intervals

	Leisure time physical activity								
	Low at 2000-02			Moderate at 2000-02			Vigorous at 2000-02		
	Low at 2007	Moderate at 2007	Vigorous at 2007	Low at 2007	Moderate at 2007	Vigorous at 2007	Low at 2007	Moderate at 2007	Vigorous at 2007
Events *n* = 264	37	23	5	27	79	15	16	29	33
Model 1	ref.	0.65 (0.39–1.10)	0.31 (0.12–0.79)	1.11 (0.72–1.72)	ref.	0.47 (0.27–0.82)	2.78 (1.53–5.05)	1.95 (1.18–3.21)	ref.
Model 2	ref.	0.70 (0.41–1.17)	0.38 (0.15–0.97)	1.05 (0.68–1.63)	ref.	0.50 (0.28–0.86)	2.42 (1.32–4.41)	1.70 (1.03–2.82)	ref.
Model 3	ref.	0.72 (0.42–1.21)	0.40 (0.16–1.03)	1.03 (0.66–1.59)	ref.	0.53 (0.30–0.92)	2.25 (1.23–4.13)	1.64 (0.99–2.81)	ref.
Model 4	ref.	0.85 (0.51–1.44)	0.44 (0.17–1.11)	1.07 (0.69–1.66)	ref.	0.60 (0.34–1.04)	2.02 (1.10–3.68)	1.61 (0.97–2.66)	ref.
Model 5	ref.	0.83 (0.49–1.40)	0.40 (0.16–1.02)	0.96 (0.62–1.50)	ref.	0.55 (0.32–0.96)	2.56 (1.40–4.68)	1.75 (1.06–2.89)	ref.

Model 1 age and gender adjusted

Model 2 age, gender, occupational social class, strenuousness of work, smoking and binge drinking adjusted

Model 3 age, gender, occupational social class, strenuousness of work, smoking, binge drinking and BMI adjusted

Model 4 age, gender, occupational social class, strenuousness of work, smoking, binge drinking and LLI adjusted

Model 5 age, gender, occupational social class, strenuousness of work, smoking, binge drinking and prior sickness absence adjusted

## Discussion

In accordance with our expectations, adopting vigorous activity was associated with a reduced risk of disability retirement over a follow-up of 6 years. Decreasing physical activity from vigorous to low or to moderate activity showed, in contrast, an increased risk of disability retirement. Adjusting for covariates, including limiting longstanding illness and prior sickness absence, somewhat attenuated the associations. However, the associations mainly remained, suggesting that in particular, vigorous physical activity may protect against disability retirement.

Our study provides further evidence on the benefits of leisure time physical activity on maintaining work ability and preventing disability retirement. The majority of previous studies have shown that leisure time physical activity is associated with a reduced risk of disability retirement [2–4], whereas some studies have shown weak or no associations [15, 26]. A previous study on changes in physical activity and mortality suggested that the reduced risk of all-cause mortality following increasing physical activity during midlife may take a decade to manifest [11]. However, according to our study some health benefits of adopting physical activity during midlife may manifest during a shorter period. These health benefits are, however, different since typical diagnostic causes of premature exit from work and mortality are, to a large extent, dissimilar. Disability retirement is mainly due to musculoskeletal and mental causes [24], while premature mortality in midlife and early old age is mostly due to cancer and cardiovascular diseases [27].

Musculoskeletal diseases and mental disorders are the main diagnostic causes of disability retirement and account for two thirds of all disability retirements in Finland [24]. Among midlife employees, musculoskeletal causes account for the majority of disability retirements, whereas among younger employees, mental disorders are the most common cause. Physical activity showed associations with both of the main diagnostic causes, but due to a relatively low number of events, the main analyses could not be stratified. In previous studies, the associations between physical activity with these main diagnostic causes of disability retirement have been largely similar [3, 4]. Physical inactivity has been associated with many musculoskeletal diseases, such as neck and low back disorders and arthrosis [28], but also with mental disorders such as depression and anxiety [29] that likely reduce work ability and may lead to disability retirement. In addition, physical activity has many other health benefits such as reduced risk of cardiovascular disease [30], and may also contribute to the reduced risk of all-cause disability retirement found among the vigorously active.

In our previous studies, adopting vigorous physical activity was also associated with other work-related health outcomes such as a lower risk of sickness absence [13, 14]. In our present study, adopting moderate and vigorous physical activity showed a dose-response association with the risk of disability retirement; however, the association did not reach statistical significance for those adopting moderately intensive physical activity. Furthermore, those decreasing physical activity from vigorous to moderate had an increased risk of disability retirement compared with the persistently vigorously active. However, decreasing the amount and intensity of physical activity may be related to health problems and although we adjusted for LLI and sickness absence, there may be some underlying health problems affecting physical activity and subsequently leading to disability retirement. Although moderately intensive activity did not markedly contribute to the risk of disability retirement, moderately intensive activity may provide some health benefits, as other studies have, for instance, demonstrated that adopting moderately intensive physical activity is beneficial for reducing the risk of mortality [10, 31]. Furthermore, for mental health, vigorous physical activity may not provide any additional benefits to lower intensity activity [14, 32]. In addition, good cardiorespiratory fitness is a key component of good functioning [33] and has been associated with a lower risk of sickness absence [34, 35], as well as disability retirement [36]. For mortality, fitness has shown stronger associations compared to physical activity, but they may both have independent beneficial effects on health [37].

### Methodological considerations

We were able to examine whether changes in leisure time physical activity over a follow-up of 6 years is associated with the risk of disability retirement within a large sample of middle-aged female and male municipal employees from various occupations. Our data on disability retirement were derived from reliable national registers. In addition, we were able to consider various covariates, including prior sickness absence, derived as well from registers. Nonetheless, there is a possibility of reverse causality since processes leading to disability retirement usually take several years and granting disability pension in Finland is typically preceded by a 12-months sickness absence period. However, as noted, in the analyses, we considered previous health status using self-reported limiting longstanding illness, as well as register-based sickness absence. Furthermore, the proportional hazards assumption was met and there was no time dependence in the examined associations. Although we included several key confounders in the analyses, residual and unmeasured confounding may still be possible, causing uncertainty with the provided estimates. It should also be noted that we adjusted for BMI, LLI and sickness absence in separate models since they may also mediate the examined associations and are thus not purely confounding factors. In addition, the relatively small number of events concerning the group increasing from low to vigorous activity may also cause uncertainty with the estimates. Information on leisure time physical activity was self-reported and lacks validation. However, self-reports have proven useful for measuring leisure time physical activity in large surveys and there is no single questionnaire that has proven superior to another [38].

There are some characteristics in our data that limit the generalizability of the results. The participants were middle-aged municipal sector employees and 80 % of them were women which reflects the gender distribution of the employees of the City of Helsinki and the Finnish municipal sector in general. We combined women and men in the analyses and thus these results are female dominated, although the associations did not differ between the genders.

## Conclusions

Our findings supported our expectations, suggesting that adopting vigorous activity during midlife is associated with a lower risk of disability retirement. In contrast, those decreasing their activity level from vigorously active were at an increased risk of disability retirement. Vigorous physical activity should be promoted among midlife employees, as a sufficient amount and intensity of physical activity may contribute to longer work careers by preventing disability retirement.

## Abbreviations

BMI: Body mass index
CI: Confidence interval
HR: Hazard ratio
LLI: Limiting longstanding illness

## References

[1] Organisation for Economic Co-operation and Development, OECD (2010). Sickness, disability and work: Breaking the Barriers: A Synthesis of findings across OECD Countries.

[2] Robroek SJ, Reeuwijk KG, Hillier FC, Bambra CL, van Rijn RM, Burdorf A (2013). The contribution of overweight, obesity, and lack of physical activity to exit from paid employment: a meta-analysis. Scand J Work Environ Health.

[3] Fimland MS, Vie G, Johnsen R, Nilsen TI, Krokstad S, Bjorngaard JH (2015). Leisure-time physical activity and disability pension: 9 years follow-up of the HUNT Study, Norway. Scand J Med Sci Sports.

[4] Lahti J, Rahkonen O, Lahelma E, Laaksonen M (2013). Leisure-time physical activity and disability retirement: a prospective cohort study. J Phys Act Health.

[5] Janssen I, Ross R (2012). Vigorous intensity physical activity is related to the metabolic syndrome independent of the physical activity dose. Int J Epidemiol.

[6] Lahti J, Laaksonen M, Lahelma E, Rahkonen O (2010). The impact of physical activity on sickness absence. Scand J Med Sci Sports.

[7] Lee IM, Paffenbarger RS (2000). Associations of light, moderate, and vigorous intensity physical activity with longevity. The Harvard Alumni Health Study. Am J Epidemiol.

[8] Samitz G, Egger M, Zwahlen M (2011). Domains of physical activity and all-cause mortality: systematic review and dose-response meta-analysis of cohort studies. Int J Epidemiol.

[9] Lahti J, Holstila A, Lahelma E, Rahkonen O (2014). Leisure-time physical activity and all-cause mortality. PLoS One.

[10] Wannamethee SG, Shaper AG, Walker M (1998). Changes in physical activity, mortality, and incidence of coronary heart disease in older men. Lancet.

[11] Byberg L, Melhus H, Gedeborg R, Sundstrom J, Ahlbom A, Zethelius B (2009). Total mortality after changes in leisure time physical activity in 50 year old men: 35 year follow-up of population based cohort. Br J Sports Med.

[12] Wolin KY, Glynn RJ, Colditz GA, Lee IM, Kawachi I (2007). Long-term physical activity patterns and health-related quality of life in U.S. women. Am J Prev Med.

[13] Lahti J, Lahelma E, Rahkonen O (2012). Changes in leisure-time physical activity and subsequent sickness absence: a prospective cohort study among middle-aged employees. Prev Med.

[14] Holstila A, Lahti J, Lahelma E, Rahkonen O. Changes in leisure-time physical activity and subsequent sickness absence due to any cause, musculoskeletal and mental causes. J Phys Act Health. 2016, in press. 10.1123/jpah.2015-0442 27144730

[15] Ropponen A, Narusyte J, Alexanderson K, Svedberg P (2011). Stability and change in health behaviours as predictors for disability pension: a prospective cohort study of Swedish twins. BMC Public Health.

[16] Lahelma E, Laaksonen M, Lallukka T, Martikainen P, Pietilainen O, Saastamoinen P (2012). Working conditions as risk factors for disability retirement: a longitudinal register linkage study. BMC Public Health.

[17] Laaksonen M, Prättälä R, Karisto A (2001). Patterns of unhealthy behaviour in Finland. Eur J Public Health.

[18] Lallukka T, Sarlio-Lahteenkorva S, Roos E, Laaksonen M, Rahkonen O, Lahelma E (2004). Working conditions and health behaviours among employed women and men: the Helsinki Health Study. Prev Med.

[19] Lahelma E, Aittomaki A, Laaksonen M, Lallukka T, Martikainen P, Piha K (2013). Cohort profile: the helsinki health study. Int J Epidemiol.

[20] Laaksonen M, Aittomäki A, Lallukka T, Rahkonen O, Saastamoinen P, Silventoinen K (2008). Register-based study among employees showed small nonparticipation bias in health surveys and check-ups. J Clin Epidemiol.

[21] Kujala UM, Kaprio J, Sarna S, Koskenvuo M (1998). Relationship of leisure-time physical activity and mortality: the Finnish twin cohort. JAMA.

[22] Ainsworth BE, Haskell WL, Whitt MC, Irwin ML, Swartz AM, Strath SJ (2000). Compendium of physical activities: an update of activity codes and MET intensities. Med Sci Sports Exerc.

[23] Fogelholm M, Kukkonen-Harjula K (2000). Does physical activity prevent weight gain--a systematic review. Obes Rev.

[24] The Finnish Centre for Pensions. The Statistical Yearbook of Pensioners in Finland [e-publication]. ISSN 1795-522X Helsinki 2015. http://www.etk.fi/wp-content/uploads/Tilasto_suomen_elakkeensaajista_2014.pdf. Accessed 27 Apr 2016.

[25] Schoenfeld D (1982). Partial residuals for the proportional hazards regression model. Biometrica.

[26] Ropponen A, Silventoinen K, Svedberg P, Alexanderson K, Koskenvuo K, Huunan-Seppala A (2011). Health-related risk factors for disability pensions due to musculoskeletal diagnoses: a 30-year Finnish twin cohort study. Scand J Public Health.

[27] Statistics Finland. Official Statistics of Finland (OSF): Causes of death [e-publication]. ISSN = 1799-5078. Helsinki 2015. http://www.tilastokeskus.fi/til/ksyyt/kat_en.html. Accessed 16 Mar 2016.

[28] Vuori IM (2001). Dose-response of physical activity and low back pain, osteoarthritis, and osteoporosis. Med Sci Sports Exerc.

[29] Ströhle A (2009). Physical activity, exercise, depression and anxiety disorders. J Neural Transm.

[30] Penedo FJ, Dahn JR (2005). Exercise and well-being: a review of mental and physical health benefits associated with physical activity. Curr Opin Psychiatry.

[31] Woodcock J, Franco OH, Orsini N, Roberts I (2011). Non-vigorous physical activity and all-cause mortality: systematic review and meta-analysis of cohort studies. Int J Epidemiol.

[32] Lahti J, Lallukka T, Lahelma E, Rahkonen O (2013). Leisure-time physical activity and psychotropic medication: A prospective cohort study. Prev Med.

[33] Huang Y, Macera CA, Blair SN, Brill PA, Kohl HW, Kronenfeld JJ (1998). Physical fitness, physical activity, and functional limitation in adults aged 40 and older. Med Sci Sports Exerc.

[34] Strijk JE, Proper KI, van Stralen MM, Wijngaard P, van Mechelen W, van der Beek AJ (2011). The role of work ability in the relationship between aerobic capacity and sick leave: a mediation analysis. Occup Environ Med.

[35] Kyrolainen H, Hakkinen K, Kautiainen H, Santtila M, Pihlainen K, Hakkinen A (2008). Physical fitness, BMI and sickness absence in male military personnel. Occup Med (Lond).

[36] Karpansalo M, Lakka TA, Manninen P, Kauhanen J, Rauramaa R, Salonen JT (2003). Cardiorespiratory fitness and risk of disability pension: a prospective population based study in Finnish men. Occup Environ Med.

[37] Blair SN, Cheng Y, Holder JS (2001). Is physical activity or physical fitness more important in defining health benefits?. Med Sci Sports Exerc.

[38] van Poppel MN, Chinapaw MJ, Mokkink LB, van Mechelen W, Terwee CB (2010). Physical activity questionnaires for adults: a systematic review of measurement properties. Sports Med.

